# The reporting of progression criteria in protocols of pilot trials designed to assess the feasibility of main trials is insufficient: a meta-epidemiological study

**DOI:** 10.1186/s40814-019-0500-z

**Published:** 2019-11-03

**Authors:** Lawrence Mbuagbaw, Sarah Daisy Kosa, Daeria O. Lawson, Rosa Stalteri, Oluwatobi R. Olaiya, Ahlam Alotaibi, Lehana Thabane

**Affiliations:** 1 Department of Health Research Methods, Evidence and Impact, McMaster University, Hamilton, ON Canada; 2 Biostatistics Unit, Father Sean O’Sullivan Research Centre, St Joseph’s Healthcare—Hamilton, 50 Charlton Avenue East, 3rd Floor Martha Wing, Room H321, Hamilton, ON L8N 4A6 Canada; 3 Centre for the Development of Best Practices in Health, Yaoundé, Cameroon; 4 Toronto General Hospital, University Health Network, Toronto, ON Canada; 5 Michael G. DeGroote School of Medicine, McMaster University, Hamilton, ON Canada; 6 Department of Pediatrics, McMaster University, Hamilton, ON Canada; 7 Departments of Paediatrics and Anaesthesia, McMaster University, Hamilton, ON Canada; 8 Centre for Evaluation of Medicine, St Joseph’s Healthcare—Hamilton, Hamilton, ON Canada; 9 Population Health Research Institute, Hamilton Health Sciences, Hamilton, ON Canada

**Keywords:** Pilot, Feasibility, Trials, Progression criteria, Protocols

## Abstract

**Introduction:**

Pilot and feasibility trials are conducted to determine feasibility or to collect information that would inform the design of a larger definitive trial. Clear progression criteria are required to determine if a definitive or main trial is feasible and how it should be designed. We sought to determine how often progression criteria are reported and the associated factors.

**Methods:**

We conducted a methodological review of protocols for pilot randomised trials published in three journals that publish research protocols (*BMJ Open*, *Trials*, *Pilot and Feasibility Studies*), using a PubMed search (2013–2017). We extracted bibliometric information including the country in which the study was conducted, source of funding, type of intervention, use of a primary feasibility outcome, sample size reporting, and justification. We used generalised linear models to determine the factors associated with reporting progression criteria.

**Results:**

Our search retrieved 276 articles, of which 49 were not eligible. We included 227 articles. Overall, 45/227 (19.8%; 95% confidence interval [CI] 14.8–25.6) reported progression criteria. Protocols published in more recent years were significantly associated with higher odds of reporting progression criteria (adjusted odds ratio [aOR] 1.40; 95% CI 1.03–1.92; *p* = 0.034). Pilot trials from Europe (aOR 0.19; 95% CI 0.08–0.48; *p* < 0.001) and the rest of the world (aOR 0.05; 95% CI 0.01–0.18; *p* < 0.003) compared to North America were significantly associated with lower odds of reporting progression criteria. Journal, source of funding, sample size, intervention type, and having a primary outcome related to feasibility were not significantly associated with reporting progression criteria.

**Conclusion:**

Progression criteria are not often explicitly stated in protocols of pilot trials leaving room for varied interpretation of findings. The development of formal guidance for progression criteria in protocols of pilot trials is warranted.

## Background

Pilot and feasibility studies are increasingly being used to inform the feasibility and design of larger trials [1]. They may be used to test procedures, instruments, and techniques that would be applied in a main study [2]. They help to provide useful information on the processes required to implement the trial, resources required, management issues, and scientific information (safety, dosing, treatment effect, etc.) [3]. Though often used interchangeably, [1] feasibility and pilot studies are not synonymous. Feasibility studies encompass the broad range of studies that address concerns about feasibility, and include randomised pilot studies, non-randomised pilot studies, and other types of non-pilot feasibility studies [4]. In this paper, we focused on small-scale randomised trials designed to inform the conduct of a future larger trial. We make no distinction on whether they are internal (integral and structurally similar to the main trial) or external (meant to provide information that will determine the structure of the main trial).

The value of pilot studies is increasingly being recognised. The UK Medical Research Council, the Canadian Institutes for Health Research (CIHR), and the US National Institutes for Health (NIH) all recommend the use of pilot studies to inform larger trials [5–7].

They are not meant to provide definitive information on treatment effects, and therefore, hypothesis testing is discouraged [1, 8]. Likewise, sample size estimates drawn from pilot studies may be misleading, given that they are often very small [3]. Many studies are pointing out concerns with how pilot studies are conducted and reported. For example, Arain et al. found that many pilot studies inappropriately focus on hypothesis testing [1]. Duffet et al. found that pilot trials in the paediatric literature focus on clinical outcomes and rarely justify their sample sizes or report criteria for success [9]. The abstracts of pilot trials in heart failure were found to be poorly reported [10]. Other authors have noted that in very few reports of pilot studies is it stated that they were conducted in preparation for a larger trial [11].

Current guidance suggests that sample size estimations for pilot studies may be done in a variety of ways depending on whether it is an internal or an external pilot study, some based on rules of thumb, the nature of the outcomes (continuous or binary), others based on the confidence interval approach or as a fraction of the fully powered large trial [3, 12, 13]. Pilot studies are generally small, but small studies should not be labelled as pilot studies if they are not pilot studies.

Given these concerns, recent efforts such as the Consolidated Standards of Reporting Trials (CONSORT) extension for pilot trials have outlined recommended approaches for reporting pilot trials [14]. As precursors to larger trials, pilot studies are expected to provide information on whether a larger trial is feasible and if so how it should be designed. Other authors have suggested strategies to select, interpret, and apply progression criteria (criteria that inform the decision to progress to a larger definitive trial) [15]. While it is expected that these criteria be reported in the pilot study manuscript, it is also important that these progression criteria be pre-specified at the protocol stage. In fact, it is recommended that these progression criteria be agreed upon by the funders and investigators [15]. Statistical approaches to informing progression have also been suggested [16]. Often, there is no detailed outline of the decision-making process that would lead to stopping, amending, or proceeding to a larger trial, [15] as such it is unclear whether the decision to continue with a larger trial was determined a priori or post hoc.

Avery and colleagues propose a traffic light system for specifying progression criteria for internal pilot studies, where green (go) indicates that the criteria have been met and the trial should proceed, amber (amend) indicates that some changes should be made to the larger trial, and red (stop) indicates that the investigators should not move forward with the larger trial [15]. Some examples of the application of progression criteria include the following: a pilot trial of strategies to enhance venous thromboprophylaxis in which the investigators deemed the trial to be definitely feasible if ≥ 70% of eligible patients completed the risk assessment form [17], and a pilot trial of rituximab for non-splenectomized patients with immune thrombocytopaenia in which the progression criteria include the recruitment of at least 60 patients in 12 months and successful blinding of staff, among others [18].

The research protocol is the ideal opportunity for investigators to report key methodological issues including the use and interpretation of progression criteria. However, there is currently no guidance on how to report a protocol for a pilot trial. In order to inform the use and interpretation of progression criteria among trialists and other stakeholders, we therefore sought to investigate the use of progression criteria in protocols of pilot trials.

We hypothesised that the use of progression criteria might be associated with certain study characteristics. For example, reporting quality improves over time, journals have different editorial policies that influence the nature of the final publication, research capacity varies by country, source of funding and study size might be indicative of the resources (including methods scientists) available to complete the study, and the type of intervention (pharmacological versus not pharmacological) may be linked to funding and may play a role in how information is reported. These study characteristics have been found to be associated with reporting in other studies [19].

## Objectives

Our objectives were to describe reporting of progression criteria to main trial and to determine the factors associated with reporting of progression criteria.

## Methods

### Design

We conducted a methodological review of protocols of pilot studies published in the past 5 years (2013–2017) in three journals known to publish research protocols: *British Medical Journal* (*BMJ*) *Open*, *Pilot and Feasibility Studies* (PAFS), and *Trials*. All three journals are indexed in PubMed. We applied the following search strategy, including terms for the journals of interest, protocol, pilot or feasibility, and time limits (01 January 2013 to 31 December 2017):

*((BMJ Open [Journal] OR Pilot Feasibility Stud [Journal] OR Trials [Journal])) AND (Pilot [Title] OR Feasibility [Title] AND Protocol [Title])*

### Data management

The full text of all identified citations was screened for eligibility. The eligibility criteria were as follows: (1) published in one of the three journals of interest, (2) a protocol for a pilot randomised trial, and (3) within the time range 2013–2017. Data were extracted by one reviewer and verified by a second independent reviewer. Agreement statistics were not captured. We extracted the following data: bibliographic information (author, year, and journal); country of origin, study objectives, main outcome measures or feasibility criteria, and presence of progression criteria; source of funding; and sample size estimation and justification for sample size.

When the planned sample size was reported as a range of values, the median was taken. When different sample sizes were reported for the different participants (e.g. health workers, patients, carers), we used the sample size for those who would be randomised. We categorised the studies as small or large, based on the median sample size of all the studies. The country in which the pilot was planned was collected and reorganised into world regions to facilitate analyses. We grouped the justifications for sample size as inadequate (based on intervention effect size, other similar studies, or no justification given) or adequate (based on a feasibility outcome, a proportion of the larger trial, and recommendations in literature).

Study data were collected and managed using the Research Electronic Data Capture (REDCap) tool hosted at St Joseph’s Healthcare Hamilton. REDCap is a secure, web-based application designed to support data capture for research studies, providing an intuitive interface for validated data entry; audit trails for tracking data manipulation and export procedures; automated export procedures for seamless data downloads to common statistical packages; and procedures for importing data from external sources [20].

## Analysis

First, data were summarised descriptively as counts (percentages) in cross tabulations according to whether they reported progression criteria.

Second, we used generalised linear models to determine the relationship between reporting of progression criteria (yes/no) and study characteristics. We assumed a binomial distribution (applying the logit link). The covariates were entered as a block: journal (*PAFS*, *BMJ Open*, and *Trials*), year of publication (continuous), source of funding (industry or government/private), sample size (small [0–60], large [> 60]), region (North America, Europe, other), primary outcome related to feasibility (yes/no), and intervention type (pharmacological versus non-pharmacological). These variables have been shown to be associated with reporting standards [19]. The level of significance was set at α = 0.05. Model fit was assessed using Akaike’s information criterion (AIC), comparing a full model with all the predictors and a reduced model with selected predictors. Crude odds ratio (OR) and adjusted odds ratios (aOR), corresponding 95% confidence intervals (CI), and *p* values are reported. Data were analysed using the *glm* command in Stata 15 (StataCorp, 2017. Stata Statistical Software: Release 15. College Station, TX: StataCorp LLC) [21].

## Results

**Fig. 1:**
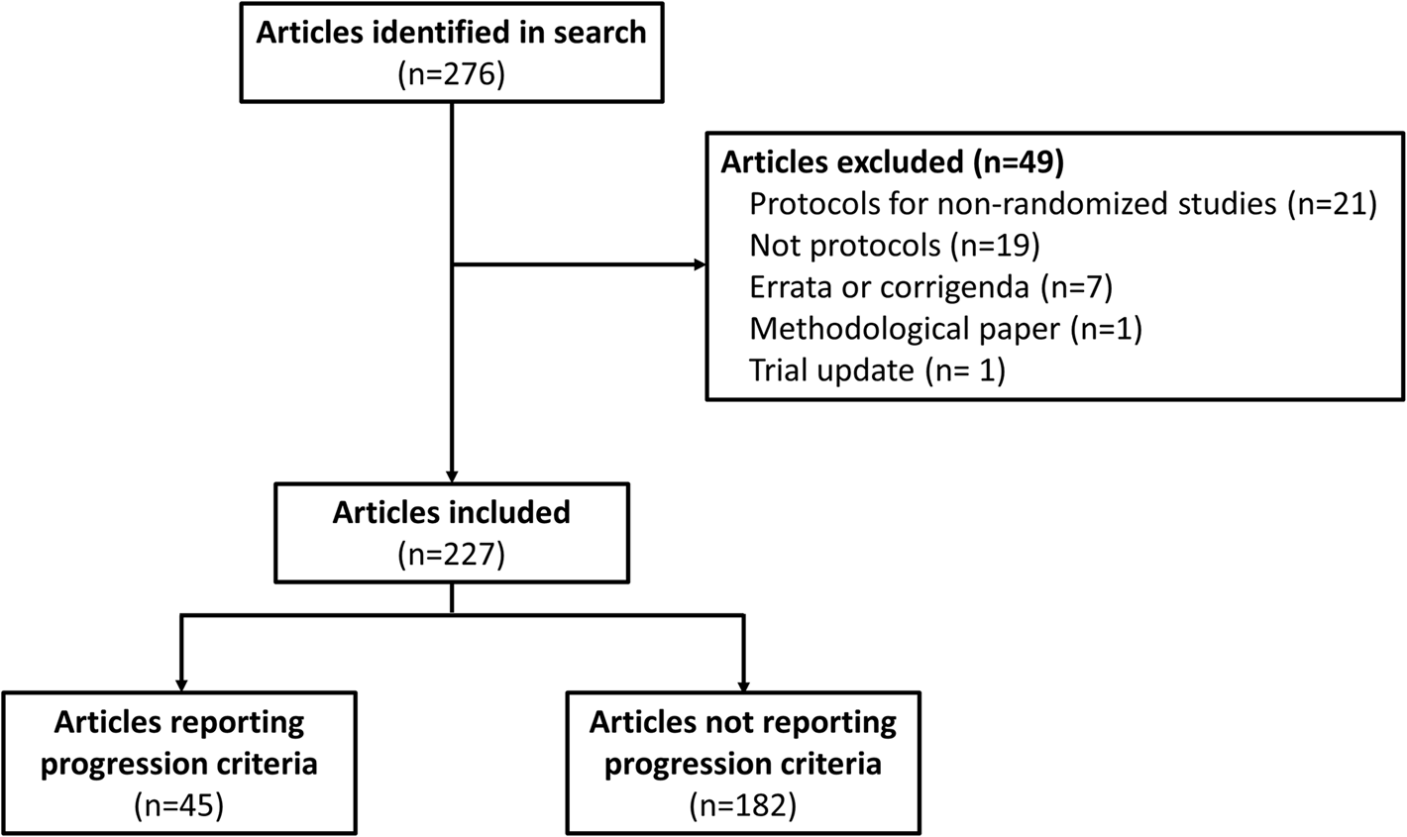
Flow chart of study selection

Our search retrieved 276 studies, of which 49 were not eligible (21 were protocols for non-randomised studies, 19 were full reports, 7 were errata or corrigenda, 1 was a methodological paper, and 1 was a trial update). Of the 227 included studies, only 45 (19.8%, 95% CI 14.8–25.6) reported progression criteria. Our screening process is outlined in Fig. 1.

Almost half (50.2%) of the studies came from *Trials* and were conducted in Europe (52.9%). More studies were published in 2017 than in any other year (31.7%). The other characteristics of the included studies are reported in Table 1.

**Table 1:** Characteristics of included studies

Variable	Number (%)
Overall	227 (100)
Journal	
PAFS*	42 (18.5)
BMJ Open**	71 (31.1)
*Trials*	114 (50.2)
Year of publication	
2013	34 (15.0)
2014	37 (16.3)
2015	34 (15.0)
2016	50 (22.0)
2017	72 (31.7)
Region	
North America	43 (18.9)
Europe	120 (52.9)
Other	64 (28.2)
Funding	
Industry	52 (24.5)
Government or private	160 (75.5)
Intervention type	
Pharmacological	35 (15.4)
Non-pharmacological	191 (84.1)
Feasibility outcomes (yes)	123 (54.2)
Sample size reported (yes)	220 (96.9)
Sample size	
Small (*n* = 0–60)	143 (64.7)
Large (*n* > 60)	78 (35.3)
Sample size justification	
Adequate	99 (44.8)
Inadequate	122 (55.2)

*Pilot and Feasibility Studies

**British Medical Journal

The proportion of studies reporting progression criteria by key study characteristics are outlined in Table 2.

**Table 2:** Distribution of studies that reported progression criteria

Variable	Number (%); 95% CI*
Overall	45 (19.8); 14.8–25.6
Journal	
PAFS**	9 (20.0); 9.6–34.6
BMJ Open***	16 (35.6); 21.9–51.2
*Trials*	20 (44.4); 29.6–60.0
Year of publication	
2013	4 (8.9); 2.5–21.2
2014	3 (6.7); 1.3–18.3
2015	5 (11.1); 3.7–24.1
2016	19 (42.2); 27.6–57.8
2017	14 (31.1); 18.2–46.7
Region	
North America	21 (46.7); 31.6–62.13
Europe	21 (46.7); 31.6–62.13
Other	3 (6.7); 1.3–18.3
Funding	
Industry	12 (28.6); 14.6–41.9
Government or private	30 (71.4); 51.1–80.0
Intervention type	
Pharmacological	7 (15.6); 6.5–29.5
Non-pharmacological	37 (82.2); 67.9–92.0
Feasibility outcomes (yes)	29 (64.4); 48.8–78.13
Sample size reported (yes)	45 (100); 92.13–100.00
Sample size	
Small (*n* = 0–60)	29 (64.4); 48.8–78.1
Large (*n* > 60)	16 (35.6); 21.9–51.2
Sample size justification	
Adequate	28 (62.2); 46.5–76.2
Inadequate	17 (37.8); 23.7–53.5

*Confidence interval for percentage

**Pilot and Feasibility Studies

***British Medical Journal

Table 3 includes the results of both unadjusted univariate and adjusted multivariable analyses (model 1 and model 2). After multivariable adjustment, more recent year of publication (adjusted odds ratio [aOR] 1.40; 95% CI 1.03–1.92; *p* = 0.034) was associated with reporting progression criteria. Pilot trials from Europe (aOR 0.19; 95% CI 0.08–0.48; *p* < 0.001) and the rest of the world (aOR 0.05; 95% CI 0.01–0.18; *p* < 0.003) compared to North America were significantly associated with lower odds of reporting progression criteria. Journal, source of funding, sample size, and having a primary outcome related to feasibility were not associated with reporting progression criteria in this model (model 1). In a reduced model (model 2), excluding source of funding, sample size, intervention type, and feasibility outcomes, articles from Europe (aOR 0.22; 95% CI 0.10–0.49; *p* < 0.001) and the rest of the world (aOR 0.04; 95% CI 0.01–0.15; *p* < 0.001) were less likely to report progression criteria. See Table 3.

**Table 3:** Factors associated with using progression criteria

Variable	Univariate model		Multivariable model 1		Multivariable model 2	
	OR (95% CI)	*p*	aOR (95% CI)	*p*	aOR (95% CI)	*p*
Journal						
*PAFS*	1		1		1	
*BMJ Open*	1.07 (0.42–2.69)	0.891	2.43 (0.68–8.61)	0.170	1.97 (0.69–5.58)	0.204
*Trials*	0.78 (0.32–1.88)	0.581	1.67 (0.49–5.56)	0.414	1.33 (0.48–3.68)	0.588
Year of publication	1.30 (1.02–1.65)	0.037	1.40 (1.03–1.92)	0.034	1.34 (1.00–1.80)	0.050
Source of funding						
Industry	1		1		Not in model	
Government or private	0.77 (0.36–1.64)	0.497	0.72 (0.26–1.99)	0.532	Not in model	
Sample size						
Small (*n* = 0–60)	1		1		Not in model	
Large (*n* > 60)	1.01 (0.51–2.01)	0.967	1.27 (0.58–2.79)	0.549	Not in model	
Region						
North America	1		1		1	
Europe	0.22 (0.08–0.62)	0.004	0.19 (0.08–0.48)	< 0.001	0.22 (0.10–0.49)	< 0.001
Other	0.05 (0.03–0.09)	< 0.001	0.05 (0.01–0.18)	< 0.001	0.04 (0.01–0.15)	< 0.001
Intervention type						
Non-pharmacological	1		1		Not in model	
Pharmacological	1.01 (0.41–2.49)	0.977	0.79 (0.27–2.36)	0.396	Not in model	
Feasibility outcomes						
Yes	1		1		Not in model	
No	0.59 (0.31–1.11)	0.100	0.95 (0.43–2.10)	0.862	Not in model	
AIC	Not applicable		0.933		0.880	

*PAFS Pilot and Feasibility Studies*, *BMJ British Medical Journal*, *OR* unadjusted odds ratio, *aOR* adjusted odds ratio, *AIC* Akaike’s information criterion

## Discussion

In this methodological review, we have shown that at the planning stages of pilot trials (i.e. in published protocols), progression criteria are not often reported, and sample sizes not often adequately justified. The use of progression criteria appears to be associated with some study characteristics of the manuscript such as journal and region of publication.

To the best of our knowledge, this is the first methodological review of protocols for pilot trials and highlights some important concerns in the design and reporting of these protocols. The small number (19.8%; 95% CI 14.8–25.6) reporting clear progression criteria is concerning, given that progression criteria are required to determine how the results of feasibility will be interpreted. If progression criteria are not set a priori, there is a risk that some studies that did not do well in the pilot stage may be moved to a larger trial without modification or due acknowledgement of potential limitations. On the other hand, successful pilot trials or trials with amenable concerns may not proceed to larger trials if interpretation of success is subjective.

We identified a few study level characteristics that were associated with the use of progression criteria. Each of these characteristics has previously been shown to be associated with reporting quality of trials in general.

Journal characteristics including endorsement of specific reporting standards and impact factor influence the nature of published reports. This has been shown in other methodologic reviews in which journals endorsing the CONSORT statement or requiring its use and higher impact factor journals published papers with better reporting [22–24]. In this study, we did not find the journal of publication to be associated with progression criteria.

As researchers develop more tailored guidance for reporting research and journals endorse these reporting standards, it can be expected that reporting will improve over time. Other studies have shown that reporting improves over time [25, 26]. More recent studies were more likely to report progression criteria only in our univariate analyses and multivariable analyses.

Larger studies tend to have better reporting quality [25, 27, 28]. Study size is probably a reflection of the resources available to complete the study. These resources would include methodological support and therefore better reporting of key methodological issues. Other studies have previously highlighted some differences when statisticians are involved, such as better interpretation of negative trials, [29] sample size calculation, and computations for multiple endpoints [30]. We did not find any association between study size and reporting of progression criteria.

The role of region in reporting of science is unclear and may have to do with differential research methods capacity and the use of English as a native language. For example, other methodological papers have shown better reporting in non-Chinese reports, compared to those from China, [31] and in North American and UK reports, compared to Scandinavia and other countries [25]. In this study, North American and European studies were more likely to have progression criteria reported.

Source of funding also influences reporting, sometimes in favour of industry-funded trials or non-industry-funded trials [27, 32, 33]. In this study, we did not find any association between funding and reporting of progression criteria.

Even though other studies have noted better reporting of pharmacological intervention studies, [34] we did not find any association.

Sample size justification in pilot studies is a subject of debate. While it is generally agreed that a calculation is not always required, there must be a reason for including a particular number of people in a study for ethical, scientific, and economic reasons. The literature includes numerous approaches to estimating sample size which we considered to all be some form of justification [12, 13, 35]. A complete absence of justification or using the sample size from a previous study were both considered inadequate.

Previous research has shown that building pilot studies around clinical or efficacy outcomes instead of feasibility outcomes (as the main outcome) is associated with worse reporting [36]. In this study, we did not find any association between having a primary feasibility outcome and reporting progression criteria. This may be because some studies reported progression criteria based on secondary feasibility criteria, even though the primary outcome was based on a clinical/efficacy outcome.

We advise some caution in the interpretation of our findings: first, because we acknowledge the relative novelty of discussions around the use of progression criteria, their importance, and how they should be reported, and second, the absence of formal guidance or reporting standards for their use. In addition, there may be other journals that publish protocols of pilot studies that we were unaware of. However, this work adds to the growing list of methodological concerns with pilot trials [1, 9–11, 36] and highlights areas for improvement. Further, there may be interaction between some of the study characteristics. For example, some journals have been publishing for longer than others, and researchers in certain regions may have preferences for certain journals. In cross tabulations, we found significant associations between journal and year (no studies from *PAFS* in 2013 and 2014; more studies from *Trials* across all years) and between journal and region (*BMJ Open* mostly publishing from the rest of the world and *Trials* mostly publishing papers from Europe). These issues could be investigated further in other studies but are unlikely to be unique to pilot studies.

As such, we recommend the development of formal guidance on the design and reporting of protocols of pilot trials, and that protocols of pilot studies clearly indicate what information will inform the decision to move to a larger trial, without which the pilot does not fulfil its purpose.

## Conclusion

Progression criteria are not often reported in protocols of pilot trials. There is room for the development of formal guidance and recommendations on the use of progression criteria in pilot randomised trials. Investigators should outline a list of feasibility criteria, how they will be interpreted, and how this interpretation will inform progression to a larger trial. The consequences of not using progression criteria, including ill-informed large trials, are enough justification to warrant a closer look at pilot studies with no explicit progression criteria.

## Data Availability

The datasets used and/or analysed during the current study are available from the corresponding author on reasonable request.

## Abbreviations

aOR: Adjusted odds ratio
*BMJ*: *British Medical Journal*
CI: Confidence intervals
CIHR: Canadian Institutes for Health Research
CONSORT: Consolidated Standards of Reporting Trials
GEE: Generalised estimation equations
NIH: National Institutes for Health
OR: Odds ratio
PAFS: Pilot and feasibility studies
REDCap: Research Electronic Data Capture
UK: United Kingdom
